# The FunGenES Database: A Genomics Resource for Mouse Embryonic Stem Cell Differentiation

**DOI:** 10.1371/journal.pone.0006804

**Published:** 2009-09-03

**Authors:** Herbert Schulz, Raivo Kolde, Priit Adler, Irène Aksoy, Konstantinos Anastassiadis, Michael Bader, Nathalie Billon, Hélène Boeuf, Pierre-Yves Bourillot, Frank Buchholz, Christian Dani, Michael Xavier Doss, Lesley Forrester, Murielle Gitton, Domingos Henrique, Jürgen Hescheler, Heinz Himmelbauer, Norbert Hübner, Efthimia Karantzali, Androniki Kretsovali, Sandra Lubitz, Laurent Pradier, Meena Rai, Jüri Reimand, Alexandra Rolletschek, Agapios Sachinidis, Pierre Savatier, Francis Stewart, Mike P. Storm, Marina Trouillas, Jaak Vilo, Melanie J. Welham, Johannes Winkler, Anna M. Wobus, Antonis K. Hatzopoulos, for the “Functional genomics in embryonic stem cells” Consortium

**Affiliations:** 1 Department of Medicine -Division of Cardiovascular Medicine and Department of Cell & Developmental Biology, Vanderbilt University, Nashville, Tennessee, United States of America; 2 Max-Delbrück-Center for Molecular Medicine (MDC) Berlin-Buch, Berlin, Germany; 3 Institute of Computer Science, University of Tartu, Tartu, Estonia; 4 INSERM U846, Stem Cell and Brain Research Institute, Bron, France; 5 BioInnovation Zentrum, Technische Universitaet Dresden, Dresden, Germany; 6 UMR 6543 CNRS, Centre de Biochimie, Nice, France; 7 Université Bordeaux 2, CNRS-UMR 5164, Bordeaux, France; 8 Max-Planck-Institute of Molecular Cell Biology and Genetics, Dresden, Germany; 9 Institute of Neurophysiology, University of Cologne, Cologne, Germany; 10 Queens Medical Research Institute E2.47, University of Edinburgh, Edinburgh, United Kingdom; 11 Sanofi-Aventis, Centre de Recherche de Paris, Paris, France; 12 Instituto de Medicina Molecular, Faculdade de Medicina de Lisboa, Lisboa, Portugal; 13 Max-Planck-Institute of Molecular Genetics, Berlin, Germany; 14 Centre for Genomic Regulation (CRG), UPF, Barcelona, Spain; 15 Institute of Molecular Biology & Biotechnology-FORTH, Heraklion, Greece; 16 Leibniz Institute (IPK), Gatersleben, Germany; 17 Department of Pharmacy and Pharmacology, Centre for Regenerative Medicine, The University of Bath, Bath, United Kingdom; 18 Institute of Clinical Molecular Biology and Tumor Genetics, German Research Center for Environmental Health, Helmholtz Center Munich, Munich, Germany; The University of Hong Kong, China

## Abstract

Embryonic stem (ES) cells have high self-renewal capacity and the potential to differentiate into a large variety of cell types. To investigate gene networks operating in pluripotent ES cells and their derivatives, the “Functional Genomics in Embryonic Stem Cells” consortium (FunGenES) has analyzed the transcriptome of mouse ES cells in eleven diverse settings representing sixty-seven experimental conditions. To better illustrate gene expression profiles in mouse ES cells, we have organized the results in an interactive database with a number of features and tools. Specifically, we have generated clusters of transcripts that behave the same way under the entire spectrum of the sixty-seven experimental conditions; we have assembled genes in groups according to their time of expression during successive days of ES cell differentiation; we have included expression profiles of specific gene classes such as transcription regulatory factors and Expressed Sequence Tags; transcripts have been arranged in “Expression Waves” and juxtaposed to genes with opposite or complementary expression patterns; we have designed search engines to display the expression profile of any transcript during ES cell differentiation; gene expression data have been organized in animated graphs of KEGG signaling and metabolic pathways; and finally, we have incorporated advanced functional annotations for individual genes or gene clusters of interest and links to microarray and genomic resources. The FunGenES database provides a comprehensive resource for studies into the biology of ES cells.

## Introduction

Stem cells hold great promise for tissue repair after injury or as a result of disease[1]. Studies in animal models and clinical trials indicate that stem cells and their progeny may replace damaged tissue improving organ recovery and function [2], [3]. For this reason, understanding the programs controlling self-renewal and differentiation of stem cells may facilitate the development of tools to unlock their regenerative potential. To this end, mouse embryonic stem (ES) cells offer an accessible and pertinent model system because they give rise to many different cell types in a reproducible manner, can be propagated practically indefinitely, have relatively stable karyotypes, and are easy to genetically manipulate [4]–[7]. Moreover, ES cell differentiation *in vitro* recapitulates events that take place during early embryonic development including the formation of the three germ layers of ectoderm, mesoderm and endoderm, and the emergence of endothelial, hematopoietic, cardiac, neuronal and hepatic or pancreatic cells [8], [9].

Functional studies have highlighted the critical roles of genes such as *Oct4* (*Pou5f1*), *Nanog* and *Sox2* in the maintenance of ES pluripotency and suppression of differentiation pathways [10]–[19]. Chromatin immunoprecipitation and chip analyses revealed that both active and silenced genes in ES cells are directly bound by one or more of these three proteins [17], [19]. The recent discoveries of new pluripotency factors including Klf4, Sall4, Zfp206, Esrrb, Tcl1, Tbx3 and Zfx suggest that the expansion and fate of ES cells follows a complex course requiring the coordinated action of a number of yet to be characterized genes [20]–[26].

The links between the many genes involved in the maintenance of pluripotency and the regulation of ES cell differentiation programs are not well characterized. Microarray studies have the potential to piece together groups of co-regulated genes and thus lead to the discovery of novel components of genetic pathways in ES cells. In recent years, a number of genome-wide approaches have identified transcripts present in mouse and human ES cells or their differentiated derivatives using a variety of gene expression profiling methods [24], [27]–[32]. This wealth of information also underscored a degree of variability and “biological noise” among data sets [33], [34].

The “Functional Genomics in Embryonic Stem Cells” consortium comprising 20 research groups (acronym FunGenES; http://www.fungenes.org) has analyzed the transcriptome of ES cells under a series of diverse stimuli during growth expansion and differentiation. Besides information gathered to answer specific experimental questions, as determined by the interests of individual partners [35]–[41], the collective data offered the opportunity to search for coordinated gene expression patterns in a systematic exploration of the mouse ES transcriptome under a battery of different experimental settings, thus minimizing possible site-specific artifacts. The results have been organized in an interactive, open-access database with a number of novel features and search tools to promote studies into the biological properties of embryonic stem cells.

## Results

### Coordinated analysis of the mouse ES cell transcriptome

The FunGenES consortium collected gene expression profiling data from mouse ES cells in a coordinated fashion by streamlining techniques and standardizing experimental protocols among partners. To this end, consortium members selected three ES cell lines (CGR8, E14TG2a and R1) for common use; ES cell clones were karyotyped and tested by alkaline phosphatase staining before being distributed to most of the consortium groups. A number of laboratories shared serum batches and used a common LIF source. Finally, RNA samples were prepared following the same procedure and subsequent microarray analyses were performed in a central facility using Affymetrix Mouse 430 v.2 arrays.

The configuration of each of the eleven individual experiments and the RNA samples collected are summarized in Table 1. In brief, the studies consisted of seven analyses on gene regulation in undifferentiated ES cells, focusing on LIF targets, Stat3 and PI3K regulated genes, as well as global gene expression changes through epigenetic mechanisms; and, four studies where ES cells were allowed to differentiate in monolayers or as embryoid bodies. Differentiation took place either in control culture media, or in the presence of various agents including retinoic acid (RA), Fibroblast Growth Factor-2 (FGF2) and Wnt pathway activators. Detailed descriptions of the individual experimental settings are included in the Supplemental File S1. The total number of tested conditions was 67, each performed in up to six, separate, biological replicates using a total of 258 Affymetrix arrays.

**Table 1:** Outline of the eleven experimental data sets in the FunGenES study.

Sample^a^	Experiment^b^	ES clone^c^	Conditions^d^	Repeats^e^
INS-2	Comparison of ES clones	E14TG2a/CGR8/R1	3	5
INS-1	Stat-3 targets in ES cells	E14TG2a	5	4–6
CNRS-UMR-5164	LIF targets in ES cells	CGR8	6	5
UOB-1	PI3-K targets in ES cells	E14TG2a	2	5
UOB-2	PI3-K targets in ES cells	E14TG2a	6	3
IMBB-1	TSA effects on ES cells	CGR8	3	3
TUD-1	Tag effects on ES cells	E14TG2a	6	3
UKOE-1	Standard ES differentiation	CGR8	9	3
AVEF-1	ES differentiation under neurogenic conditions	E14TG2a	11	4^f^
CNRS-UMR-6543	ES differentiation under adipogenic conditions	CGR8	9	3
IPK-1	ES differentiation favoring the pancreatic lineage	R1	7	5

a experiment abbreviation.

b short description of individual experiment.

c ES cell line used in the corresponding experiment.

d number of different conditions analyzed.

e number of independent replicates.

f four replicates except AVEF-1 eb4 (two repeats).

Comparison of gene expression profiles showed a low number of differentially expressed transcripts among the three ES cell lines. Using 5% false discovery rate in ANOVA calculation in any of the 3 comparisons (CGR8 vs. E14TG2a vs. R1), there are 137 genes (0.9% of the analyzed transcripts) that show a 2-fold difference or higher in expression levels among the three lines; 34 of these genes are >2-fold higher or lower expressed in E14TG2a, 5 in CGR8 and 11 in R1 cells.

### Organizational design and special features of the FunGenES database

**Figure 1:**
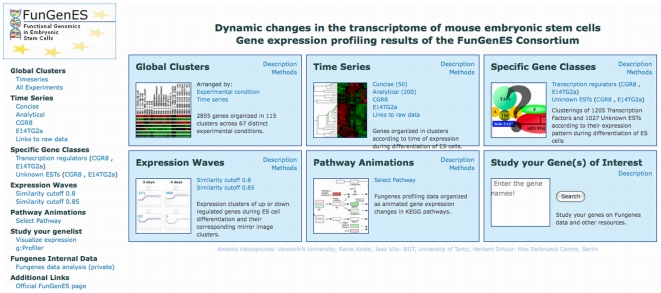
Outline of the FunGenES database. The database home site is separated in six parts. The upper row views provide entry to different ways the FunGenES data sets have been organized: in clusters using the entire 67 experimental conditions (Global Clusters, top left); according to time of expression (Time Series) in 50 (Concise) and 200 (Analytical) clusters using a subset of experimental samples representing conditions without additional stimuli (top middle); or depending on gene class, i.e., transcriptional regulators or ESTs (Specific Gene Classes, top right). In Time Series and Specific Genes Classes, the expression profiles at various differentiation time points are also provided separately for CGR8 and E14TG2a cells. The bottom row consists of three tools that offer: detailed organization of specific gene expression patterns (Expression Waves) during the differentiation process (bottom left); animation of KEGG pathways organized by successive days of differentiation (Pathway Animations, bottom middle); and, a search engine to obtain the expression of any transcript or groups of transcripts in the Affymetrix 430 v.2 arrays during the ES cell differentiation process (bottom right). In all entry points, buttons provide a “Description” of how the data have been organized and the “Methods” used to construct the various tools.

To enhance the analytical power of the collected information, facilitate data mining and provide public access of the consortium results to the scientific community, the expression data have been organized in an open, interactive database (http://biit.cs.ut.ee/fungenes/) with a number of original features and tools (Figure 1). These include: a) **Global Clusters** that consist of a small, tight subset of genes that are co-expressed under the entire spectrum of experimental conditions; b) **Time Series** of gene expression profiles during successive days of standard ES cell differentiation; c) **Specific Gene Classes** based on hierarchical clustering of transcriptional factors and ESTs; d) **Expression Waves** of genes with characteristic expression profiles during ES cell differentiation, juxtaposed to waves of genes that behave in the exact opposite way; e) **Pathway Animations** that illustrate dynamic changes in the components of individual KEGG signaling and metabolic pathways viewed in time-related manner; and, f) **Search Engines** to display the expression pattern of any transcript, or groups of transcripts, during the course of ES cell differentiation, or to query the association of candidate genes with various FunGenES database clusters. In addition, there are cross-links to annotate and characterize these genes in the context of other relevant genomic and stem cell resources.

Gene expression profiles are provided for all RNA samples combined, or separately for the CGR8 and E14TG2a ES cell lines. The list of genes belonging to a cluster together with the heatmaps of individual transcripts, appear by clicking on the corresponding cluster. The heatmaps of gene clusters or single genes can be displayed in different color codes or configured using a range of analytical parameters using the ExpressView tool. With subsequent marking of any gene, or groups of genes, it is possible to zoom in to the clustering visualization. In addition, when a subset of genes is selected, it is possible to access functional analysis and other relevant resources via the URLMAP link aggregator. This provides crosslinks to external resources such as NCBI Entrez, Ensembl, iHOP, Pubgene, MEM - Multi Experiment Matrix, and a number of genomics and stem cell databases. There is also a link to the g:Profiler tool that provides functional annotation to assess the biological classification of transcripts with specific expression patterns [42]. Terms of description in g:Profiler include GO categories [43], KEGG [44] and Reactome pathways [45], miRBase microRNA information [46], and TRANSFAC motifs [47]. In addition to functional explanations, g:Profiler provides convenient tools for dealing with different gene identifiers and finding orthologs from other organisms.

### Identification of gene sets with similar expression profiles across all tested experimental conditions

The synchronized genomic analyses among consortium partners presented the opportunity to search for coordinately expressed genes, either during ES cell differentiation, or in response to various stimuli. Towards this goal, we mined the genomics data to identify sets of genes, the expression of which performed in the same way over the entire spectrum of experimental conditions.

In order to facilitate the interpretation of the bioinformatic output, and enhance the biological significance of the computational data, we pre-selected probe sets corresponding to previously characterized genes. The initial focus on known genes with common expression profiles across many conditions, allowed us to interpret differences between conditions, as well as to identify specific core groups of genes that could serve as anchor-points for mapping gene function in future analyses. Specifically, we applied exclusion criteria to screen out transcripts without annotation and of unknown origin, as well as hypothetical transcripts or proteins. This selection reduced the number of transcripts from 45,101 to 32,020. We then removed redundant probe sets, and probe sets that showed minor differences in expression levels across all tests setting the standard deviation of the log_2_ signal over 67 conditions to less than 0.45. The selection criteria brought the number of transcripts used for cluster analysis to 5,959.

**Figure 2:**
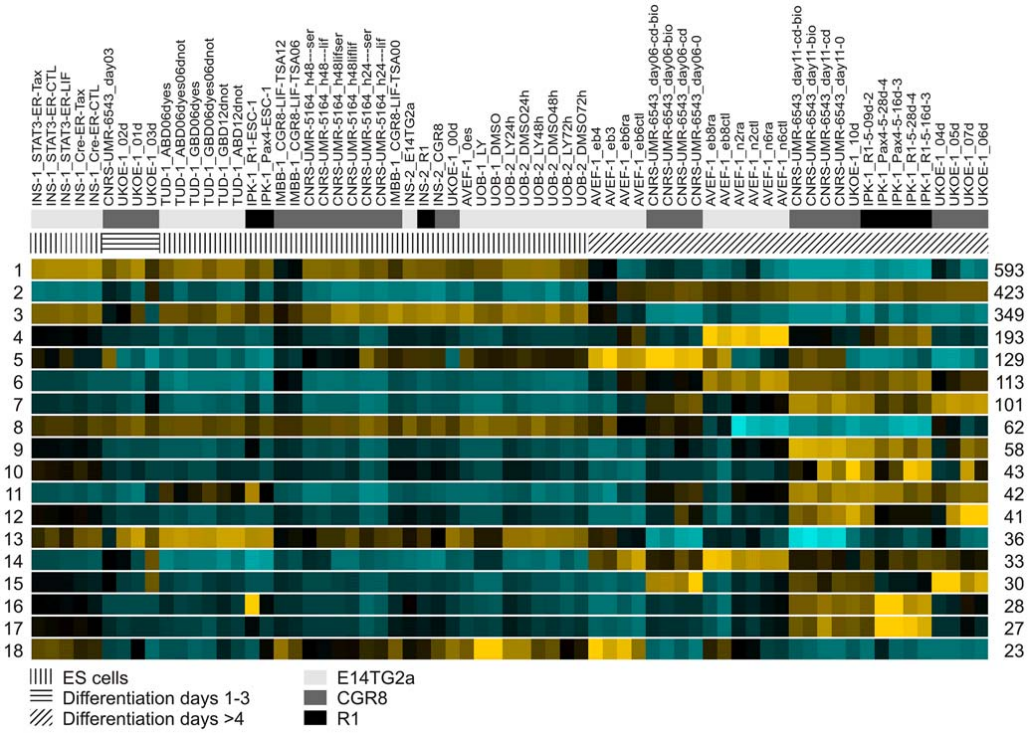
Global hierarchical clustering analysis of the FunGenES microarray data. The average heatmaps of the eighteen largest clusters with at least 20 members are displayed. Hierarchical clustering organized samples according to differentiation stages, with undifferentiated ES cells and early differentiation states at the left and progressively more differentiated states toward the right. The cluster number is depicted on the left side, the cluster size, i.e., the number of transcripts in the cluster, is shown on the right. Heatmap colors range from cyan (very low or no expression) over black (low/middle) to yellow (high expression levels). The names of the 67 RNA samples are indicated on the top. The description of the acronyms and the experimental conditions for each sample are provided in Table 1 and in Supplemental File S1, respectively. The names of the ES cell clones used in each experiment and the time of RNA isolation during the ES cell differentiation process are coded with bars above the heatmaps. Explanatory notes of the codes are indicated below the heatmaps.

Unsupervised hierarchical clustering of the 5,959 genes, using 100 random permutations, gave rise to 115 groups, containing a total number of 2,855 transcripts, with a probability of 95% or higher that clustering was not random (Supplemental File S2). Eighteen clusters had >20 transcripts, fifteen clusters contained between 10–19 transcripts, whereas the remaining eighty two clusters had 3–9 members. The heatmaps of the eighteen largest clusters with >20 transcripts are shown in Figure 2. The heatmaps and the complete list of genes belonging to each cluster, ordered by cluster size, are available in Supplemental File S3 and in the FunGenES database under the heading “Global Clusters”.

**Figure 3:**
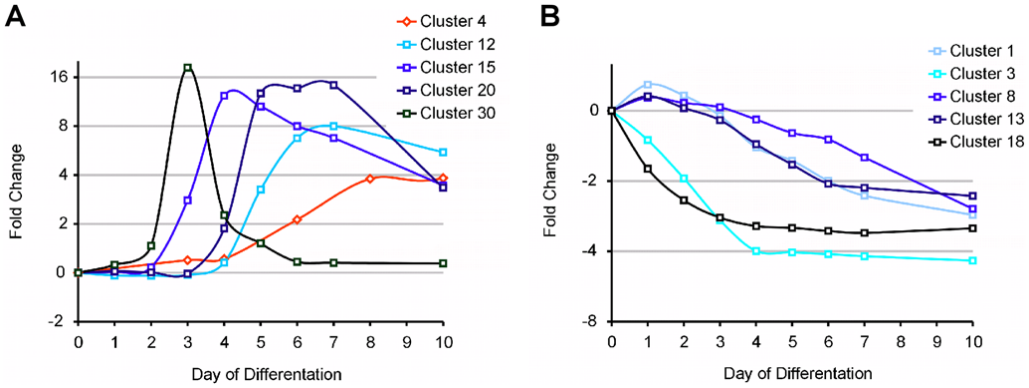
Average expression profiles of selected Global Clusters. A. Average expression levels of Global Clusters 4, 12, 15, 20 and 30 that contain up regulated transcripts during ES cell differentiation. The clusters consist of genes specific for gastrulation (cluster 30), mesoderm formation (15), hemopoiesis (20), cardiopoiesis (15) and neurogenesis (4). The sequential appearance of genes specific to early developmental stages matches the timing of their induction during embryonic development. B. Average expression levels of all the transcripts in Global Clusters 1, 3, 8, 13 and 18 containing genes the expression of which decreases upon differentiation. Expression in Clusters 3 and 18 is suppressed early, at the onset of differentiation, whereas the expression of genes in clusters 1, 8 and 13 declines in gradual fashion. Clusters 1, 3, 8, 13 and 18 include genes that take part in cell cycle, proliferation and metabolism, as well as in self-renewal and maintenance of ES cell pluripotency.

The functional annotations of all the clusters with ≥10 transcripts, which were obtained using the on GO classification categories of the g:Profiler tool for all the genes in each cluster, are shown in Table 2 (for downregulated genes during ES cell differentiation) and Table 3 (for upregulated genes). Inspection of the data illustrates that in many instances, hierarchical clustering grouped genes that have been functionally associated with particular developmental and/or cellular processes. For example, clusters containing genes that are upregulated during the course of ES cell differentiation (Table 3) include in order of time of expression: cluster 30 that represents genes which take part in the formation of the three embryonic germ layers during gastrulation, i.e., *Goosecoid*, *Cerberus like 1 homolog*, *Wnt3*, *Mesp1*, *Mixl1*, *mEomes* and *Even-skipped 1*; cluster 15 containing molecular regulators of early mesoderm development including *Bmp2*, *Bmp5*, *Msx1*, *Msx2*, *Cripto*, *Tbx20*, *Hey2*, *Smad6*, *Vegfr2* (*Kdr*), *Foxf1* and *Hand1*; cluster 20, which comprises regulatory and structural genes linked to hemopoiesis such as *Gata1*, *Nfe2*, *Klf1*, *Tie1*, hemoglobins (*Hba-x*, *Hbb-b1*) and *Glycophorin A*; cluster 12, which is rich in genes involved in cardiac development, e.g., *Mef2c*, *Myl4*, *cardiac Troponin T2*, *Tropomodulin 1*, *myosin binding protein C*, *Bves, Angiopoietin 1* and *Angiopoietin 2*; and, cluster 4, which consists mostly of genes associated with neuronal development and differentiation, for example, *Neurog1*, *Neurog2*, *Olig2*, *Nkx6.1*, *Neurod4*, *Pou3f2*, *Pou3f4*, *Cacna2d3*, *Cacng4*, *Kcnq2* and *EphA5*. The average expression pattern of all the genes in these clusters is depicted in Figure 3A.

**Table 2:** Functional annotation of Global Clusters of downregulated genes^a^.

Cluster	Gene Number	Functional Annotation
1	593	Cellular Machinery: Cell Cycle, Organelles Nucleic Acid Metabolism, Synthesis and Processing
3	349	Transcriptional Regulation, Metabolism
8	62	Cell Cycle, Organelles, Nucleic Acids metabolism and binding
13	36	Cell Cycle, Signal transduction, Nucleic Acids metabolism, Transcription
18	23	Cell Cycle, Meiosis
21	14	No strong annotation: Signal Transduction
24	13	Ribosomal proteins
25	13	Cell Cycle, Replication
27	12	No strong annotation
28	12	t-RNA biosynthesis
33	10	No strong annotation

a clusters containing 10 or more genes that are downregulated during ES cell differentiation.

**Table 3:** Functional annotation of Global Clusters of upregulated genes^a^.

Cluster	Gene Number	Functional Annotation
2	423	Development, Morphogenesis, Signal transduction, Apoptosis
4	193	Neurogenesis, Development, Morphogenesis
5	129	No strong annotation
6	113	No strong annotation: Cell adhesion, Signal transduction, Neuronal
7	101	Cardiovascular development, Branching morphogenesis, extracellular matrix
9	58	Extracellular matrix biosynthesis, Cell adhesion
10	43	Immune response
11	42	Extracellular matrix biosynthesis
12	41	Cardiovascular development
14	33	Development, Morphogenesis, Transcriptional regulation
15	30	Mesoderm development, Branching morphogenesis, Vascular development
16	28	Extracellular matrix, Collagen pathway
17	27	Extracellular matrix, Chemokines, Cell adhesion
19	18	No strong annotation: Cell proliferation, immune response
20	17	Hematopoiesis
22	14	No strong annotation: Signal Transduction
23	13	No strong annotation
26	13	No strong annotation
29	11	No strong annotation
30	10	Gastrulation, Cell migration
31	10	Calcium binding, structural proteins
32	10	No strong annotation

a clusters containing 10 or more genes that are upregulated during ES cell differentiation.

Taking into account that ES cells are isolated at embryonic day 3.5 post fertilization, the sequential appearance of genes specific for gastrulation, mesoderm formation, hemopoiesis, cardiopoiesis and neurogenesis during ES cell differentiation follows the timing of comparable developmental stages in embryonic development. For example, the transient expression of cluster 30 genes at day 3 *in vitro*, which corresponds to embryonic day E6.5 (3.5+3), matches the expression timing of genes such as *Cerberus-like 1* and *Wnt3 in vivo* [48], [49]. In a similar manner, the induction of hematopoietic (cluster 20, day 3.5+4 = E7.5) and cardiovascular-specific (cluster 12, 3.5+5 = E8.5) genes follows the chronological order of the appearance of blood islands and the formation of the heart tube during embryonic development [50], [51].

In contrast to the complex induction scheme of clusters representing upregulated genes, clusters containing genes that decrease upon differentiation form fewer clusters that fall mainly in two categories, of genes suppressed early, at the onset of differentiation (clusters 3 and 18), and of genes downregulated in more gradual fashion (clusters 1, 8 and 13; Figure 3B). Downregulated clusters include mostly genes that take part in cell cycle, proliferation and metabolism, as well as genes that have been implicated in the maintenance of ES cell pluripotency (Table 2). For instance, Cluster 1 contains genes such as *cyclin A2*, *cyclin B1*, *cyclin E1*, *cyclin F*, *polymerase alpha 2*, *RNA polymerase II polypeptide H*, and *RNA polymerase III polypeptide G*, whereas Cluster 3 genes include *Nanog*, *Sox2*, *Pou5f1* (*Oct4*), *Klf2*, *Zpf42* (*Rex1*) and *Esrrb*.

The validation rate of the microarray expression profiling data was 90.7%, based on results obtained independently in eleven consortium laboratories. In brief, 330 of 364 genes, tested by quantitative or conventional PCR, gave comparable expression patterns to the data obtained by microarray analysis. A representative comparison of expression profiles obtained by Q-PCR and array analysis for fifteen genes, belonging to five of the clusters depicted in Figure 3, is shown in Supplemental File S4.

Since there is a higher than 95% chance that cluster assignments are accurate (Supplemental File S2), and our validation analysis shows that 90.7% of the array expression patterns match the RNA analysis results using other techniques (e.g., Q-PCR), we estimate that more than 86% of the genes in a cluster follow the corresponding average expression profile. It is likely that these genes are components of related molecular or cellular pathways, or they might be targets of common regulatory mechanisms, or both [52]–[54]. Next to well-characterized genes, clusters often contain transcripts the function of which is poorly understood. Our analysis predicts that the latter participate in the same biological processes as the known genes in the corresponding clusters – thus providing a starting point to study the function of poorly characterized transcripts.

### Time Series and Specific Gene Classes of the FunGenES Database

**Figure 4:**
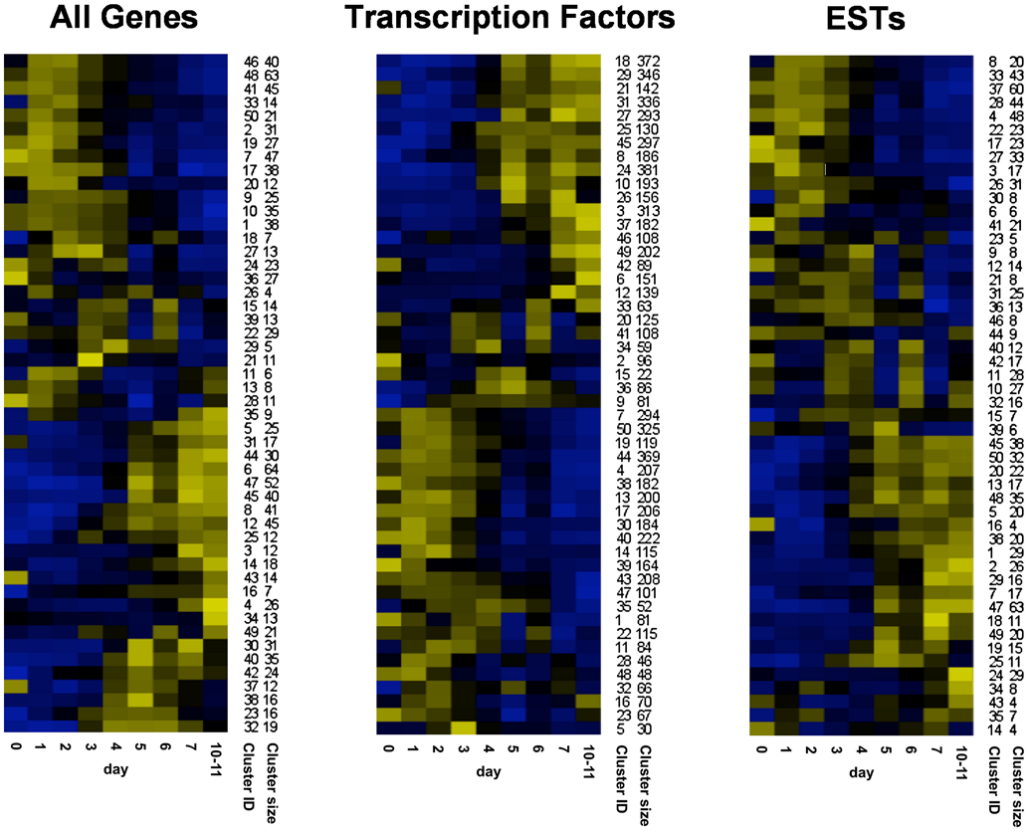
Time Series and Specific Gene Classes of the FunGenES database. Heatmaps of the 50 concise clusters for All Genes, Transcription Factors and ESTs according to their timing of induction or suppression during the normal ES cell differentiation process. Day 0 represents undifferentiated ES cells, days 1–7 and 10–11, the corresponding differentiation days. The first column to the right of each cluster is the cluster number (cluster ID); the second column is the number of genes that belong to the cluster (cluster size).

To better visualize changes in gene expression programs during differentiation, we performed *k*-means clustering analysis, followed by hierarchical clustering, to group genes by their timing of induction or suppression during the normal ES cell differentiation process. For this purpose, we used the data from a subset of consortium samples representing untreated states without additional stimuli (26 conditions listed in Supplemental File S5) and, we included transcripts with significant differential expression among samples (standard deviation>0.45). The resulting “Time Series”, containing 8,211 genes, have been organized in 50 Concise (Figure 4) and 200 Analytical clusters.

The “Time Series” clusters expanded the number of genes that follow a specific expression pattern revealed in the previous global hierarchical clustering. For example, cluster 5 of the concise “Time Series”, which consists of transiently induced genes around day 3 of differentiation, similarly to Global Cluster 30, contains the same transcripts, but in addition, it also includes *T-brachyury*, *Axin2*, *Mesp2*, *Fgf8*, *Wnt8a*, *Sp5*, *Sp8*, *Follistatin*, *Mix1* and *Lim1*. These genes have been also implicated in the gastrulation phase of embryogenesis [55] indicating that “Time Series” clusters provide a comprehensive collection of genes expressed at specific stages of ES cell differentiation and early embryonic development.

To assist searches of interconnected circuits of gene expression regulators, we carried out clustering of genes related to transcriptional activation (Figure 4). Finally, we analyzed ESTs separately to distinguish the ones that are expressed in ES cells or during the differentiation process. From approximately 12,000 ESTs, only 1,027 show a specific expression pattern (8.6% of all ESTs present in the Affymetrix 430 2.0 microarray). This is in contrast to known genes where 21% have a particular pattern, possibly because a number of ESTs included in the microarrays are cloning artifacts. However, the remaining 1,027 ESTs might represent novel transcripts with potentially important functions in stem cell biology and embryonic development. The 1,027 ESTs have been grouped in 50 clusters based on their timing of appearance (Figure 4). About half are expressed specifically in ES cells, the rest in ES cell derivatives. Transcription factor and EST clusters can be accessed through the “Specific Gene Classes” window of the FunGenES Database.

### Gene “Expression Waves”

**Figure 5:**
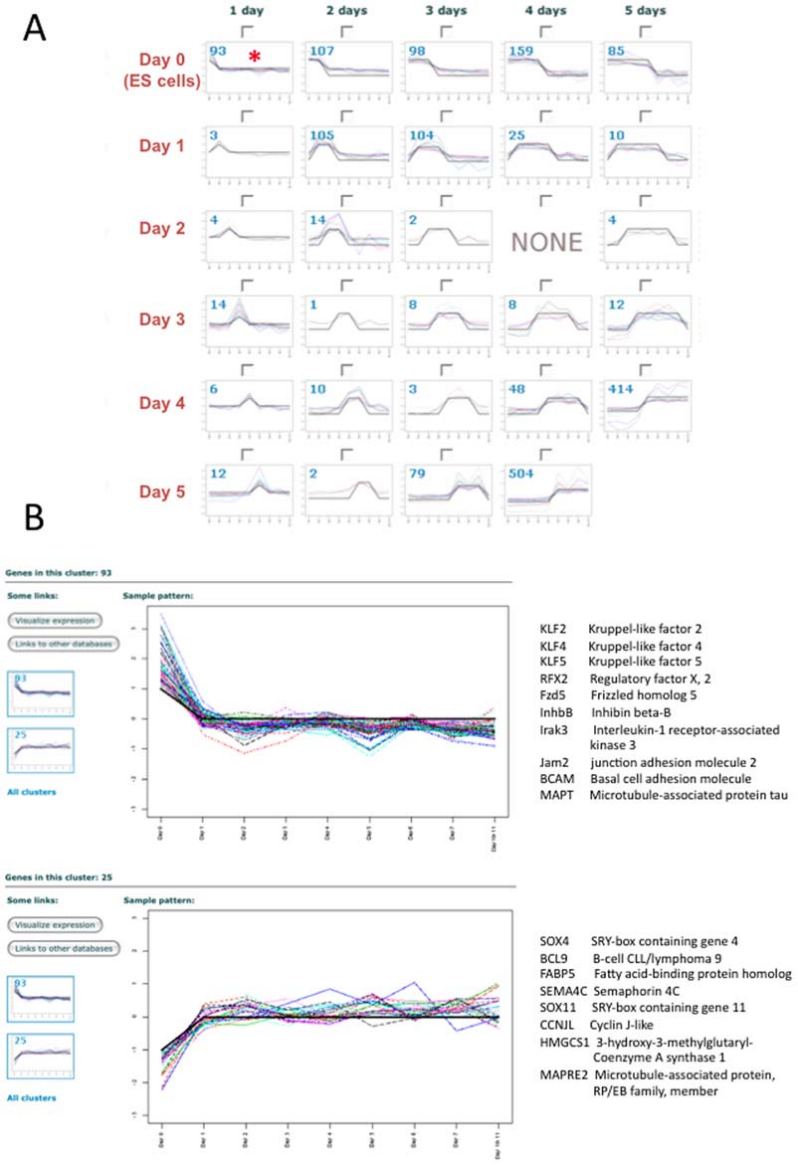
The “Expression Waves” tool of the FunGenES database. A. Partial snap shot of the “Expression Waves” window that depicts detailed specific expression patterns of genes during consecutive days of ES cell differentiation. Numbers in individual panels represent the number of genes the expression of which matches the specific profile. For example, the top left panel (red asterisk) represents 93 genes that become down regulated as soon as differentiation begins. After clicking on any of the panels, for example on the top left panel marked with a red asterisk, the expression profiles of the individual genes that follow the corresponding pattern as well as their names appear in a new window (as shown in *B*). B. Individual expression profiles of the 93 genes marked with a red asterisk in *A* are shown in the top panel. The graph and gene content representing transcripts expressed in the opposite manner is available on the same page for side-by-side comparison (lower graph on the left). Clicking on this graph opens a new window with the corresponding genes (bottom panel). Representative examples of genes belonging in the depicted Expression Waves are shown on the right of the graphs. Using the two link buttons on the left, it is possible to further study the gene list of a particular “wave” in the context of publicly available databases, or view the gene expression profiles in ExpressView.

To better illustrate and map co-regulated genes with different activation and deactivation profiles, the levels of every transcript have been assigned to graphs of “Expression Waves” that follow a particular, predetermined, expression pattern (Figure 5). The names of genes belonging to the corresponding “Expression Wave” are included below the graph. The graph and gene content representing transcripts expressed in the opposite manner is available on the same page for side-by-side comparisons. In this way, it is possible to search the database for groups of potentially interconnected genes as a starting point to decipher regulatory networks of transcription factors, signaling molecules and membrane receptors, or for indications of genes that might be co-regulated by the same genetic pathways.

### Search Engines of the FunGenES database and links to external databases

**Figure 6:**
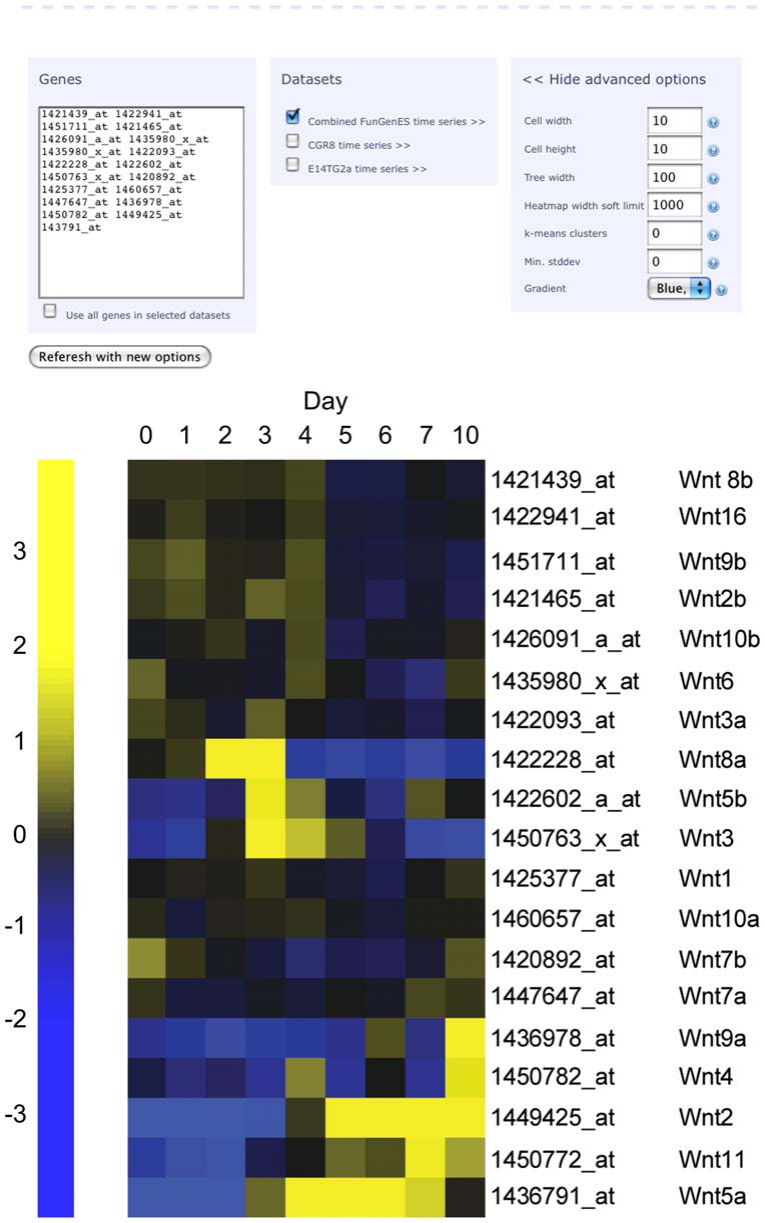
The FunGenES database Search Engine for gene expression profiles during ES cell differentiation. Expression profiles of the 19 Wnt genes during ES cell differentiation with the corresponding Affymetrix IDs using the ExpressView feature. Wnts have grouped in members expressed during early differentiation stages (Wnt2b, Wnt3a, Wnt6, Wnt8b, Wnt9b, Wnt10b, Wnt16); transiently around day 3 of differentiation (Wnt3, Wnt5b, Wnt8a); or, Wnt genes that appear primarily after day 4 (Wnt2, Wnt4, Wnt5a, Wnt7a, Wnt7b, Wnt9a, Wnt11). The heatmaps show weak expression of Wn1 and Wnt10a throughout differentiation and high levels of Wnt7b in undifferentiated ES cells. Besides Affymetrix IDs, searches can be performed with any standard gene name or identifier, as well as by mixing ID types. The expression profiles in different ES cell lines can be obtained by selecting the corresponding dataset. A number of options (top right) allow custom configuration of data analysis and presentation.

To maximize the analytical power of the database and integrate it with the existing genomic and stem cell resources, we included the “Study your Gene(s) of Interest” search engine. For any gene of interest, or group of genes, it provides via URLMAP links to display the expression profile across the entire spectrum of the FunGenES data. This provides electronic analysis of the expression profile of any gene(s) in mouse ES cells and during the subsequent stages of differentiation by using standard abbreviated gene names, Affymetrix probe set IDs, or any identifier supported by the Ensembl database. An example of the expression profiles for the 19 members of the Wnt protein family of morphogens in ES cells and during the first 10 days of differentiation, obtained using the FunGenES search engine, is shown in Figure 6. The search tool also provides a fast assessment of expression profiling data obtained by RT-PCR or other techniques. The design allows easily addition of future data sets to expand and update the analytical power of the search engines.

In addition to the visualization of expression profiles during ES cell differentiation, the search engine provides links to analyze the selected genes using many publicly available tools and resources. As mentioned above, such links include external resources such as NCBI Entrez, Ensembl, iHOP, Pubgene, MEM - Multi Experiment Matrix, and a number of genomics and stem cell databases. Moreover, the g:Profiler toolset provides functional annotation.

### Pathway Animations

To examine the action of individual pathways *in toto* during ES cell differentiation, the FunGenES database was given an additional feature called “Pathway Animations” that depict dynamic changes in specific genetic, signaling or metabolic pathways viewed in time-related animations based on the KEGG annotation [44], [56]. The resource also offers a set of tools that allow the users to reanimate the graphs by selecting specific time points and/or subsets of pathway components.

**Figure 7:**
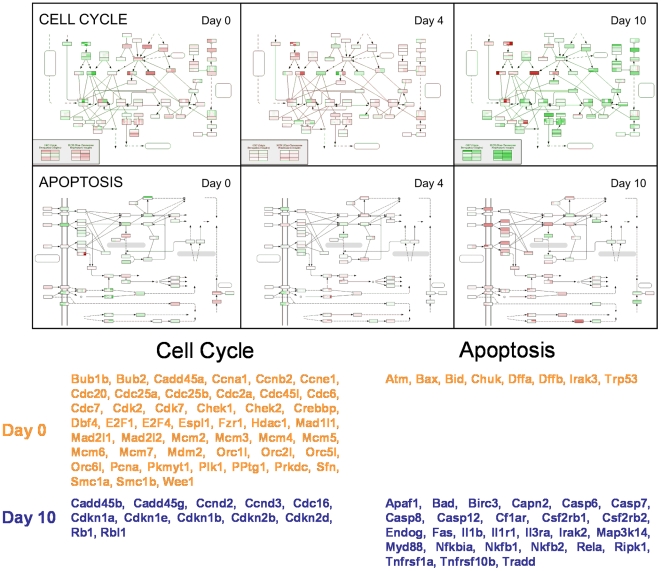
Snap shots from the animated Cell Cycle and Apoptosis KEGG pathways. The images depict day 0 (undifferentiated ES cells), day 4 and day 10 of differentiation. Each box in a pathway box represents a gene or gene family that can be visualized by marking the box during animation. The different family members are represented with juxtaposed vertical rectangles within the same box. For genes having multiple probe sets in the Affymetrix microarray, the corresponding gene rectangle is divided horizontally with each line depicting the expression level of an individual probe set. Red color marks expressed genes and green color indicates absence of detectable expression. Color intensity reflects expression levels. The table below lists the genes from the Cell Cycle and Apoptosis KEGG pathways that are expressed at day 0 and day 10. ORC: origin of replication complex; MCM: mini-chromosome maintenance.

Figure 7 depicts a stationary view of the KEGG pathways for “Cell Cycle” and “Apoptosis” at three time points; it appears that ES cells (day 0) have higher numbers of expressed genes involved in cell cycle (rectangles in red color) compared to differentiated cells (day 10). Almost all of the genes expressed at day 0 have been silenced by day 10 (green) and replaced by a new set of genes. The extensive changes in the expression profile from ES cells (day 0) to differentiated cells at day 10 are suggestive of a broad overhaul of the self-renewal machinery. The “Cell Cycle” animated pathway shows that genes encoding regulators of DNA replication are expressed at high levels in pluripotent, self-renewing ES cells and are progressively down regulated during differentiation. They include genes of the origin of replication complex (*orc*), the minichromosome maintenance (*mcm*), and the cell division cycle (*cdc*) families. Genes involved in DNA damage control and inhibition of DNA synthesis [49], in particular *Atm*, *Chk1* and *Chk2*, are also highly expressed in ES cells, but decline during differentiation. These changes are indicative of the active replication machinery and the tightly controlled replication fidelity in proliferating ES cells [57]–[59].

Undifferentiated ES cells are also characterized by elevated levels of transcripts encoding the G1/S transition-promoting complex cyclin E1:Cdk2 and, by contrast, low levels of transcripts encoding D-type cyclins and Cdk4/6 inhibitors of the INK4 family (p15, p16, p18, p19; Figure 7). Differentiation is associated with a decrease in cyclin E1 and a concomitant elevation in D-type cyclins and cdk inhibitor transcripts [57], [60]. These results are likely due to the progressive switch from a cyclin E-based autonomous cell cycle, which characterizes self-renewing ES cells, to the D-type cyclins/Retinoblastoma (Rb) protein-regulated somatic cell cycle [61].

Conversely, few pro-apoptotic genes are expressed in ES cells (day 0; most boxes appear in green), but many are gradually induced during the differentiation process showing the exact opposite pattern from the genes involved in cell proliferation (Figure 7). As observed for cell-cycle genes, there is minimal overlap between apoptosis-associated genes expressed at days 0 and 10. This strikingly complementary pattern suggests a reciprocal interrelationship between the balance of pro- and anti-apoptotic genes in ES cells and their differentiated progeny.

## Discussion

Functional analyses using loss-of-function and protein-protein interaction approaches, as well as bioinformatics tools, have began to piece together the regulatory networks active in ES cells [24], [62]. Furthermore, genome-wide studies, combining chromatin immunoprecipitation (ChIP) and array hybridization (ChIP-on-chip), have revealed that both active and silenced genes are directly bound in ES cells by one or more of the core pluripotency factors Oct4, Sox2 and Nanog [17], [19], [63].

However, it appears that the actual core factor set regulating pluripotency and early differentiation in ES cells is larger and more highly interconnected than previously suspected. Kim et al. have performed a genome-wide analysis of target promoters for nine transcription factors, namely Oct4, Sox2, Klf4, c-Myc, Nanog, Dax1, Rex1, Zpf281, and Nac1 [64]. They found that target promoters bound by a single or few factors tend to be inactive or repressed, whereas promoters bound by more than four factors are active in the pluripotent state and become repressed upon differentiation. Interestingly, targets of Myc or Rex1 are implicated in protein metabolism, whereas targets of the other factors are enriched in genes involved in developmental processes. The results also established a hierarchy within the key pluripotency factors such that Klf4 serves as an upstream regulator of feed-forward circuits involving Oct4, Sox2, Nanog and Myc.

The increasing complexity of gene regulatory networks emerging from these studies, combined with the surging amount of genomics and proteomics work, underscore the need for resources that would enable the scientific community to readily mine available and prospective data. The FunGenES database provides such a template with a number of tools including Animation of KEGG Pathways, Expression Waves, Time Series, Specific Gene Classes, such as ESTs and transcription factors, and searches for the expression pattern of any gene or transcript during ES cell differentiation using standard gene names and IDs. Search results are linked to: comprehensive annotation tools using the g:Profiler tool, which includes the presence of common regulatory motifs in promoter areas and miRNA targeting information; and, to available resources such as NCBI Entrez, Ensembl, etc.

Genomic studies, which in principle group together co-regulated genes, can potentially identify new components of known regulatory pathways in ES cells that can subsequently be explored in functional studies. In addition to well-described genes, clusters often contain transcripts the function of which has not yet been associated with a specific biological process thus providing novel unexplored links to known molecular pathways.

Although the database described here was based on the gene expression profiling results of the FunGenES consortium, it can be easily adapted to incorporate available or future genomics data obtained in ES cells. Moreover, the analytical paradigms and expression pattern clusters presented here could provide a scaffold for comparative analyses with human ES cell lines. This information will be particularly important for future evaluation of ES-like induced pluripotent stem (iPS) cells reprogrammed from somatic tissues that can be potentially used to derive pancreatic cells, cardiomyocytes or neurons for organ regeneration [21], [65]–[67]. For example, the g:Profiler tool provides the possibility to convert mouse Affymetrix probe set numbers to any Affymetrix probe set numbers from other organisms, allowing gene profiling comparisons among data sets generated in different species. This tool also allows conversion of previous Affymetrix probe set numbers (i.e., the first generation of Affymetrix microarrays - U74v2) to the more recent microarray probe set numbers (like the MG430v2 used in this study).

During the last years, a growing number of repositories of microarray data and other forms of gene expression profiles for stem cell research have been developed [68]–[71]. Data presentation is heterogeneous and ranges from: simple storage of expression data and experiment information (StemDB); presentation of lists of specific regulated transcripts (HESC); specific analysis results of a closed dataset (SCDb); or storage and visualization of variable resources with correlative and mutual information about single transcripts (one to many relationship, StemBase) [68]–[70]. To facilitate data comparison between the FunGenES database and other resources, we have included a series of links to other Stem cell databases, i.e., to SCDb, Amazonia in the Study your Gene(s) of Interest search engine. This way it is possible to obtain and compare the expression pattern of genes in the FunGenES database to the expression profiles in other tissues, experimental settings, or different stem cells types.

In contrast to existing microarray database resources, the FunGenES database includes a state of the art tools for the interactive visualization of gene to gene relationships. It provides gene lists and hierarchical matrices using co-expression analysis by distance-base clustering (k-means, hierarchical clustering), as well as integrated gene expression analyses by mapping observed gene expression changes onto specific signaling and metabolic pathways. We expect that not only regenerative medicine applications, but also basic science studies will benefit from the resources described here, especially when compared to expression profiling data obtained from loss- and gain-of-function approaches [19], [27], [72]. Furthermore, the assignment of ESTs and genes to specific pathways provide a fresh collection of novel components that can be further explored in functional assays during embryonic development and in human diseases.

## Materials and Methods

### RNA isolation and microarray hybridization

Total RNA was isolated using the RNeasy Mini Kit from Qiagen and treated with RNase-free DNase I (5 units/100 µg of nucleic acids, Sigma). Biotinylated cRNA was prepared according to the standard Affymetrix protocol [73]. In brief, double-stranded cDNA was synthesized from 10 µg total RNA using the SuperScript Choice System (Invitrogen) and the Affymetrix T7-(dT)_24_ primer. Following phenol/chloroform extraction and ethanol precipitation, the cDNA was transcribed into biotin-labeled cRNA using T7 polymerase (Ambion MEGAScript T7). cRNA products were purified using the RNeasy kit (Qiagen) and fragmented to an average size of 30–50 bases according to Affymetrix recommendations. 15 µg of fragmented cRNA were used to hybridize to the Mouse Genome 430 version 2.0 Array for 16 hrs at 45°C. The arrays were washed and stained in the Affymetrix Fluidics Station 450 and scanned using the Affymetrix GeneChip Scanner 3000 7G. The image data were analyzed with the GeneChip® Operating Software (GCOS) 1.4 using Affymetrix default analysis settings. Arrays were normalized by the log scale robust multi-array analysis (RMA) [74].

We used 258 Affymetrix GeneChips to analyze 67 individual experimental conditions (outlined in Table 1). A detailed description of the individual experiments is provided in Supplemental File S1. The eleven microarray data sets have been annotated in a MIAME compliant manner and deposited in EBI ArrayExpress (http://www.ebi.ac.uk/microarray-as/ae/. The accession numbers are as follows: AVEF-1: E-TABM-669, CNRS-UMR-5164: E-TABM-667, CNRS-UMR-6543: E-TABM-668, IMBB-1: E-TABM-670, INS-1: E-TABM-562, INS-2: E-TABM-671, IPK-1: E-TABM-493, TUD-1: E-TABM-675, UKOE-1: E-TABM-672, UOB-1: E-TABM-673, UOB-2: E-TABM-674).

Each array was checked for general assay quality (3′-5′ ratio of Gapdh <1.5, noise (RawQ) <4 and scaling factor at a TGT value of 200 <4). The robust multi-array average (rma) normalization (background-adjustment, quantile normalization and median polish summarization) has been performed using RMAExpress version 1.0 beta 4. In addition, we assessed data integrity by calculating Pearson correlation z-values over the complete dataset of 45,101 probe sets. The difference between array to array correlation within biological replicates (z = 2.73±0.38) and between non replicates (z = 1.90±0.35) indicates that there is a sufficiently high signal to noise ratio.

### Comparison of gene expression profiles in the three ES cell lines

For comparison analysis, from the 45,101 probe sets represented on the Mouse 430 version 2 array, we selected 30,526 gene-associated transcripts (eliminating transcripts without annotation and of unknown origin, as well as hypothetical transcripts or proteins). In addition, for genes represented multiple times on the microarray, we selected the transcript with the strongest average signal as representative for the respective gene. This brought the number of analyzed transcripts to n = 15,263. A 5% false discovery rate in ANOVA calculation and a 2-fold difference or higher in any of the 3 comparisons (CGR8 vs. E14TG2a vs. R1) led to a set of 137 differentially expressed genes (0.9% of the analyzed transcripts).

### Data preparation for unsupervised hierarchical clustering – Global Clusters

The first step in our data analysis was to average the biological replicates for each of the 67 experimental conditions. To identify genes that cluster together under the tested conditions, we excluded probe sets with a standard deviation in expression values of < log2 (0.45) from the vector mean. We then removed redundant gene/probe sets taking into account the ENTREZ, Unigene and RefSeq gene-id annotations. Among redundant probe sets, we selected the probe set with the highest average expression signal. We also removed probe sets of unknown origin, for example RIKEN sequences, or hypothetical transcripts/proteins. These criteria led to a data set of 5,959 transcripts.

### Unsupervised hierarchical clustering

Correlation of differentially expressed transcripts was detected by hierarchical clustering of expression values with the Cluster version 2.11 software [52] applying mean centering and normalization of genes and arrays before the computational clustering analysis. Average linkage hierarchical clustering of the data was carried out as described [75].

### Permutations

The correlation significance of expression profiles between probe sets was assessed empirically by one hundred rounds of random permutations. For each round, the 67 values for each probe set were randomly redistributed [76] and data sets clustered as described [75]. The best similarity scores of each permuted and clustered data set was collected to evaluate the 95th percentile of significant clusters in the original data set. 5% of the permuted data sets gave rise to clusters containing no more than two genes at a similarity score >0.85071 (Supplemental File S6). Clusters with 3 or more genes (115 clusters) were documented and selected for further analysis.

### Clustering of the data in Time Series

Besides the clustering described above that was based on the entire spectrum of experimental conditions, expression data were clustered according to timing of expression in a two-step strategy. First, probe sets were clustered with *k*-means into a small number of clusters using chord distance (Euclidean distance over vectors normalized to unit sphere). In a second step, the resulting clusters, represented by mean profiles, were clustered using average linkage hierarchical clustering with Pearson correlation distance measure, and visualized in a heatmap representation [52]. No filtering besides removing genes with low variation was applied to these data sets.

### Expression Waves

We developed a method to identify all genes that have characteristic expression patterns during ES cell differentiation. In brief, transcripts were included into a particular expression wave represented by a single artificial template, if its correlation with the specific pattern was higher than a certain threshold and also highest among all other patterns. This analysis was done in two different stringent conditions with correlation thresholds of 0.8 and 0.85. The results are presented in a series of graphs, with the list of genes that belong in the depicted pattern identified below. Each graph is juxtaposed to its “mirror image”, i.e., the graph representing genes that behave exactly the opposite way.

### Pathway Animations

We designed animations of pathways in the Kyoto Encyclopedia of Genes and Genomes (KEGG) [44]. The animations use diagrams available at the KEGG webpage, which portray connections between pathway components. The expression levels of relevant genes are shown in the diagrams by the standard red (high) – green (low) color codes. In case a gene family represents a particular pathway step, the corresponding box displays the expression patterns of all individual members of the family in adjacent vertical stripes. Each stripe may be further divided horizontally depicting the expression patterns of different probe sets corresponding to the same transcript.

### “Study your Gene(s) of Interest”

This feature has been designed to allow investigators to search and display the expression of any probe set during ES cell differentiation based on the FunGenES data sets. The program draws clustered heatmaps with the columns annotated with relevant sample information. The search engine recognizes common gene identifiers; the conversion to Affymetrix probe set IDs is done using Ensembl BioMart [77] mappings. The heatmap representation is based on the ExressView tool, which is linked to the URLMAP, to provide further analysis options for selected genes. The organization of the various FunGenES tools is depicted in Supplemental File S7.

## Supporting Information

Supplemental File S1 Detailed overview of the microarray experimental designs & Contact Information
(0.24 MB PDF)

Supplemental File S2 Yield of the unsupervised hierarchical clustering. Histogram of the number of clusters (y-axis) for each cluster size (x-axis). Clusters with more than 100 genes are listed separately on the top right corner.
(0.58 MB TIF)

Supplemental File S3 Gene content of the 115 Global Clusters
(0.40 MB XLS)

Supplemental File S4 Comparison of gene expression profiles obtained by Q-PCR (left panels) and microarray analysis (right panels). The gene name is depicted on top of the Q-PCR graph; the Affymetrix ID of the same gene marks the corresponding adjacent graph. CT: Cycle Threshold values of the Q-PCR analysis. Signal: normalized log2 signal values from the microarray analysis. Genes are organized according to the Global Cluster they belong as indicated. The results show comparable gene expression profiles between microarray and Q-PCR data.
(0.97 MB TIF)

Supplemental File S5 Experimental data sets used in Time Series
(0.05 MB PDF)

Supplemental File S6 Evaluation of significant correlations. Ranked plot of the best similarity scores (y-axis) of 100 permutated and clustered datasets (x-axis) and the evaluated 95th percentile of significant clusters (blue line). The results are given for cluster nodes with more than two (black line) or three (red line) cluster members.
(0.55 MB TIF)

Supplemental File S7 Schematic representation of the FunGenES Database depicting tools to view expression data sets and links to external resources and databases. Tools in boldface have been developed specifically for the FunGenES Database.
(0.79 MB TIF)
