# Supplementary material for: The FunGenES Database: A Genomics Resource for Mouse Embryonic Stem Cell Differentiation
Source: PLoS One. 2009 Sep 3;4(9):e6804. doi: 10.1371/journal.pone.0006804 (PMC2731164; doi:10.1371/journal.pone.0006804)
Supplement: Supplemental File S1 — Detailed overview of the microarray experimental designs & Contact Information (0.24 MB PDF) [file pone.0006804.s001.pdf]

Supplemental File S1

Detailed overview of the microarray experimental designs

& Contact Information

Project INS-2: comparison of three different ES cell lines

Cell lines: CGR8; E14TG2a; R1

Pierre-Yves Bourillot & Pierre Savatier

Contact: Pierre Savatier

INSERM U846

Stem Cell and Brain Research Institute

18 Avenue Doyen Lépine

69500 Bron, France

Tel: +33 4 72913442

Email: pierre.savatier@inserm.fr

Summary of the project

In order to identify differences in gene expression profiles between the three mouse ES cell lines used within the FunGenES consortium, RNA was prepared from undifferentiated CGR8, E14TG2a and R1 cells.

Abbreviation	Description	Arrays
CGR8	ES cell line CGR8	5
E14TG2a	ES cell line E14TG2a	5
R1	ES cell line R1	5

INS-2 raw data (Affymetrix cel files of type Mouse 430_2) have been stored in the EMBL EBI ArrayExpress database:

http://www.ebi.ac.uk/arrayexpress/experiments/E-TABM-671

Accession: E-TABM-671

Project INS-1: Identification of Stat3 target genes

Cell lines: E14TG2a; E14(T)-Stat3-ER; E14(T)-Cre-ER

Irène Aksoy & Pierre Savatier

Contact: Pierre Savatier

INSERM U846

Stem Cell and Brain Research Institute

18 Avenue Doyen Lépine

69500 Bron, France

Tel: +33 4 72913442

Email: pierre.savatier@inserm.fr

Summary of the project

The objective of this study is to identify target genes of the STAT3 transcription factor in ES cells, making use of the hormone-dependent STAT3-ER^{T2} protein. STAT3-ER^{T2} is a fusion protein between STAT3 and the mutated ligand-binding domain of the human estrogen receptor. In the absence of ligand, STAT3-ER^{T2} is sequestered in the cytoplasm in an inactive state by association of the ligand-free ER^{T2} domain with the ubiquitous Hsp90 complex. In the presence of tamoxifen, STAT3-ER^{T2} is liberated and translocates into the nucleus. We made use of the pPCAGIZ vector and episomal supertransfection in E14/T cells to express STAT3-ER^{T2}.

After electroporation, cells were plated at $5x10^5$ cells per 10 cm culture dish in medium supplemented with 1,000 U/ml of LIF and cultured in the presence of 1 µg/ml of zeocine for 7 days. Resistant colonies were pooled and further propagated in selection medium for 10 days in the presence of 10 nM tamoxifen and in the absence of LIF.

STAT3-ER^{T2}-expressing ES cells do not require LIF for propagation in the undifferentiated state. They are strictly dependent upon tamoxifen for self-renewal. RNAs have been prepared from: (*i*) ES cells starved of LIF and tamoxifen for 24 hours; (*ii*) starved cells after re-stimulation with 1,000 U/ml of LIF for 1 hour; and, (*iii*) starved cells after re-stimulation with 100 nM of tamoxifen for 2 hours.

E14/T cells expressing Cre-ER^{T2}, a fusion protein between the Cre recombinase and the ER^{T2} domain have been also generated and used as control cells to identify -and eliminate off-target effects of STAT3-ER^{T2}. Cre-ER^{T2}-expressing ES cells require LIF for propagation in the undifferentiated state. RNA have been prepared from: (*iv*) ES cells starved of LIF and tamoxifen for 24 hours; and, (*v*) starved cells after re-stimulation with 100 µM tamoxifen for 2 hours.

Abbreviation	Description	Arrays
STAT3-ER-CTL	STAT3-ER not stimulated	4
STAT3-ER-Tax	STAT3-ER stimulated with Tax	6
STAT3-ER-LIF	STAT3-ER stimulated with LIF	5
Cre-ER-CTL	Cre-ER not stimulated	5
Cre-ER-Tax	Cre-ER stimulated with Tax	5

INS-1 raw data (Affymetrix cel files of type Mouse 430_2) have been stored in the EMBL EBI ArrayExpress database:

http://www.ebi.ac.uk/arrayexpress/experiments/E-TABM-562

Accession: E-TABM-562

Project CNRS-UMR-5164: Identification of LIF target genes

Cell line: CGR8

Marina Trouillas and Hélène Boeuf
Contact: Hélène Boeuf
UMR 5164-CNRS- CIRID
Université Bordeaux 2
Bat. 1B, BP14
146 rue Léo Saignat
F-33076 Bordeaux Cedex, France
Tel: +33 5 57574633
Email: helene.boeuf@u-bordeaux2.fr

Summary of the project

The purpose of these experiments was to identify genes the expression of which is regulated by LIF in ES cells. To this end, CGR8 ES cells were grown in DMEM medium with 10% FCS with or without 20 ng/ml recombinant human LIF.

Five independent total RNA samples (Qiagen column) have been prepared from each cell growth condition. The proper LIF regulation has been confirmed by monitoring LIF-dependent activation of STAT3 using western blot with phospho-Tyr705 STAT3 on protein lysates prepared in parallel with each RNA preparation. The quality of RNA preparations was checked by RT-PCR with known LIF-dependent genes such as *Socs3*, *Klf4* and *JunB* (with *Hprt* serving as a non-regulated constant control).

Abbreviation	Description	Arrays
h24---ser	CGR8 cells grown in DMEM/10%FCS, without LIF for 24h have been re-induced for 25 minutes with DMEM/10%FCS	5
h24---lif	CGR8 cells grown in DMEM/10%FCS, without LIF for 24h have been re-induced for 25 minutes with DMEM/10%FCS/LIF	5
h48---ser	CGR8 cells grown in DMEM/10%FCS, without LIF for 48h have been re-induced for 25 minutes with DMEM/10%FCS.	5
h48---lif	CGR8 cells grown in DMEM/10%FCS, without LIF for 48h have been re-induced for 25 minutes with DMEM/10%FCS/LIF	5
h48lifser	CGR8 cells grown in DMEM/10%FCS/LIF for 48h have been re-induced for 25 minutes with DMEM/10%FCS	5
h48liflif	CGR8 cells grown in DMEM/10%FCS/LIF for 48h have been re-induced for 25 minutes with DMEM/10%FCS/LIF	5

CNRS-UMR-5164 raw data (Affymetrix cel files of type Mouse 430_2) have been stored in the EMBL EBI ArrayExpress database:

http://www.ebi.ac.uk/arrayexpress/experiments/E-TABM-667

Accession: E-TABM-667

Project UOB-1/UOB-2: Identification of PI3-K target genes

Cell line: E14TG2a

Mike P. Storm and Melanie Welham

Contact: Melanie Welham

Department of Pharmacy and Pharmacology

Centre for Regenerative Medicine

The University of Bath

Bath, BA2 7AY, UK

Tel: +44 1225 386428

Email: prsmjw@bath.ac.uk

Summary of the project

Aim: To identify genes regulated by PI3K signaling in ES cells.

E14tg2a cells cultivated in KO-DMEM supplemented with Knock-out serum replacement (Invitrogen) in the presence of LIF were either: (i) starved for 24h in media lacking LIF, pre-incubated for 30 minutes with 5 μM LY294002 in DMSO or DMSO alone and then treated for 2h with LIF, prior to preparation of RNA samples (UOB-1 samples); or, (ii) cultured in the continuous presence of LIF, plus DMSO alone or 5 μM LY294002 for 24 to 72h prior to RNA extraction (UOB-2 samples).

UOB-1:

Abbreviation	Description	Arrays
DMSO	DMSO + LIF 2h	5
LY	DMSO + LY294002 + LIF 2h	5

UOB-1 raw data (Affymetrix cel files of type Mouse 430_2) have been stored in the EMBL EBI ArrayExpress database:

http://www.ebi.ac.uk/arrayexpress/experiments/E-TABM-673

Accession: E-TABM-673

UOB-2:

Abbreviation	Description	Arrays
DMSO24h	24h DMSO	3
DMSO48h	48h DMSO	3
DMSO72h	72h DMSO	3
LY24h	24h DMSO+LY294002	3
LY48h	48h DMSO+LY294002	3
LY72h	72h DMSO+LY294002	3

UOB-2 raw data (Affymetrix cel files of type Mouse 430_2) have been stored in the EMBL EBI ArrayExpress database:

http://www.ebi.ac.uk/arrayexpress/experiments/E-TABM-674

Accession: E-TABM-674

Project IMBB-1: Identification of genes targeted by the histone deacetylase inhibitor Trichostatin A

Cell line: CGR8

Efthimia Karantzali and Androniki Kretsovali
Contact: Androniki Kretsovali
Institute of Molecular Biology & Biotechnology - FORTH
1527 Vassilika Vouton
GR-711 10 Heraklion, Crete, Greece
Tel: +30 2810 391191
Email: kretsova@imbb.forth.gr

Summary of the project

Aim: To investigate the effects of global histone deacetylation inhibition in ES cell differentiation.

CGR8 cells were cultivated in presence or absence of Trichostatin A (TSA, an HDAC inhibitor) for 0 (control), 6 and 12 hours, respectively.

Abbreviation	Description	Arrays
CGR8-LIF-TSA00	ESCs grown in presence of LIF and treated with TSA (HDAC inhibitor); 0 hours	3
CGR8-LIF-TSA06	ESCs grown in presence of LIF and treated with TSA (HDAC inhibitor); 6 hours	3
CGR8-LIF-TSA12	ESCs grown in presence of LIF and treated with TSA (HDAC inhibitor); 12 hours	3

IMBB-1 raw data (Affymetrix cel files of type Mouse 430_2) have been stored in the EMBL EBI ArrayExpress database:

http://www.ebi.ac.uk/arrayexpress/experiments/E-TABM-670

Accession: E-TABM-670

Project TUD-1: TAg effects on DOX MIB and DOX DEX induced clones

Cell line: E14TG2a

Sandra Lubitz**,** Konstantinos Anastassiadis and Francis Stewart
Contact: Francis Stewart
Technische Universitaet Dresden
BioInnovationZentrum
Am Tatzberg 47
01307 Dresden, Germany
Tel: +49 351 46340129
Email: francis.stewart@biotec.tu-dresden.de

Summary of the project

The purpose of the experiment was to see if changes in the expression profile of the cells caused by transient expression of T-Antigen can be reversed. Two different E14TG2a lines (ABD and GBD*) were generated: both lines carry a construct containing the SV40 Large-T Antigen under the control of the tetracycline promoter. The ABD line also contains the reverse tet-repressor fused to VP16 and Androgen Binding Domain (ABD) under the control of the CAGGs promoter. The GBD*-clone carries a construct containing the reverse tet-repressor fused to VP16 and mutated Glucocorticoid Binding Domain (GBD*) under the control of the CAGGs promoter (GBD*-clone).

The term "induction" means that the cells were treated for 6 days with either Mibolerone and Doxycycline (for the ABD clone) or with Dexamethasone and Doxycycline (for the

GBD* clone). This also signifies that the T-Antigen is expressed. “Deinduction” means that the cells were treated for 6 days with the above compounds and then cultured for an additional 6 days without the compounds. During the “deinduction” phase the expression of T-Antigen stops. Untreated cells means that the cells were cultured for the period of 12 days without any compounds, there is no expression of T-Antigen.

Abbreviation	Description	Arrays
ABD12dnot	clone 1 (ABD) 12 days untreated	3
ABD06dyes06dnot	clone 1 (ABD) 6 days induced and afterwards 6 days deinduced	3
ABD06dyes	clone 1 (ABD) 6 days induced	3
GBD12dnot	clone 2 (GBD) 12 days untreated	3
GBD06dyes06dnot	clone 2 (GBD) 6 days induced and afterwards 6 days deinduced	3
GBD06dyes	clone 2 (GBD) 6 days induced	3

TUD-1 raw data (Affymetrix cel files of type Mouse 430_2) have been stored in the EMBL EBI ArrayExpress database:

http://www.ebi.ac.uk/arrayexpress/experiments/E-TABM-675

Accession: E-TABM-675

Project UKOE-1: Differentiation of ES cells to Embryoid Bodies

Cell line: CGR8

Michael Xavier Doss, Johannes Winkler, Jürgen Hescheler and Agapios Sachinidis

Contact: Agapios Sachinidis

Institute of Neurophysiology

University of Cologne

Robert-Koch-Str. 39

50931 Cologne, Germany

Tel: +49-221-4787373

Email: a.sachinidis@uni-koeln.de

Summary of the project

CGR8 cells were cultivated as embryoid bodies (EBs) without addition of selective reagents in hanging drop cultures (day 0-7) in a time course experiment. After 7 days, EBs were plated in gelatin-coated 6-well plates. RNA was prepared from ES cells (day 0), EBs (day 1 to 7) and plated EBs (day 10).

Abbreviation	Description	Arrays
00d	ES cell line CGR8 at day 0	3
01d	ES cell line CGR8 at day 1	3
02d	ES cell line CGR8 at day 2	3
03d	ES cell line CGR8 at day 3	3
04d	ES cell line CGR8 at day 4	3
05d	ES cell line CGR8 at day 5	3
06d	ES cell line CGR8 at day 6	3
07d	ES cell line CGR8 at day 7	3
10d	ES cell line CGR8 at day 10	3

UKOE-1 raw data (Affymetrix cel files of type Mouse 430_2) have been stored in the EMBL EBI ArrayExpress database:

http://www.ebi.ac.uk/arrayexpress/experiments/E-TABM-672

Accession: E-TABM-672

Project AVEF-1: EB formation and differentiation into neuronal cells

Cell line: E14TG2a/Sox1TV2

Olivier Féraud, Murielle Gitton and Laurent Pradier

Contact: Laurent Pradier

Sanofi-Aventis

Centre de recherche de Paris

13, Quai Jules Guesde - BP 14

F-94403 Vitry sur Seine Cedex, France

Tel: +33 1 58933885

Email: Laurent.Pradier@sanofi-aventis.com

Summary of the project

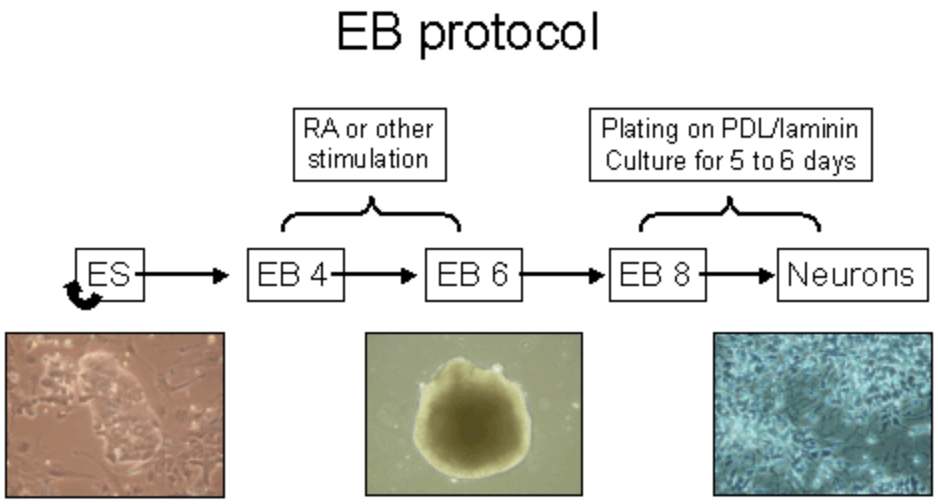

ES cells have been allowed to form embryoid bodies in liquid culture condition onto non-adherent dishes. This step takes place during 8 days with a 48 hours retinoic acid

stimulation between days 4 and 6. During this step samples have been prepared from ES cells, 3, 4, 6 and 8 days old embryoid bodies (ES, EB3-8) with (RA) or without (CTL) retinoic acid from day 6. At day 8 of differentiation EB have been dissociated and cells have been plated on poly-D-lysine/laminin coated dishes. During the first 48 hours of plating, bFGF has been added to the culture medium in order to increase the number of neuronal progenitor cells. Samples have been prepared at the end of this step (N2 for two days neurons). After removal of bFGF, cells are allowed to differentiate during 4 more days and the last samples have been prepared from this step (N6). RNA was prepared from four independent experiments and verified by RT-PCR of reference genes.

Abbreviation	Description	Arrays
0es	ES cells	4
eb3	3 days old embroid bodies	4
eb4	4 days old embroid bodies	2
eb6ctl	6 days old embroid bodies; without retinoic acid stimulation	4
eb6ra	6 days old embroid bodies; with retinoic acid stimulation	4
eb8ctl	8 days old embroid bodies; without retinoic acid stimulation	4
eb8ra	8 days old embroid bodies; with retinoic acid stimulation	4
n2ctl	(8 +) 2 days old neurons; without retinoic acid stimulation	4
n2ra	(8 +) 2 days old neurons; with retinoic acid stimulation	4
n6ctl	(8 +) 6 days old neurons; without retinoic acid stimulation	4
n6ra	(8 +) 6 days old neurons; with retinoic acid stimulation	4

AVEF-1 raw data (Affymetrix cel files of type Mouse 430_2) have been stored in the EMBL EBI ArrayExpress database:

http://www.ebi.ac.uk/arrayexpress/experiments/E-TABM-669

Accession: E-TABM-669

Project CNRS-UMR-6543: Differentiation to early mesoderm (day3) and further to adipocyte precursors (day 6)

Cell line: CGR8

Nathalie Billon and Christian Dani

Contact: Christian Dani

UMR 6543 CNRS

Centre de Biochimie

Parc Valrose

06108 Nice Cedex, France

Tel: +33 4 92076436

Email: dani@unice.fr

Summary of the project

Embryoid Bodies have been treated either with CD2314 (a Retinoic Acid Receptor β agonist), Bio (a canonical wnt pathway inducer) or both between day 3-6 of differentiation and RNA samples have been prepared at day 6 and at day 11.

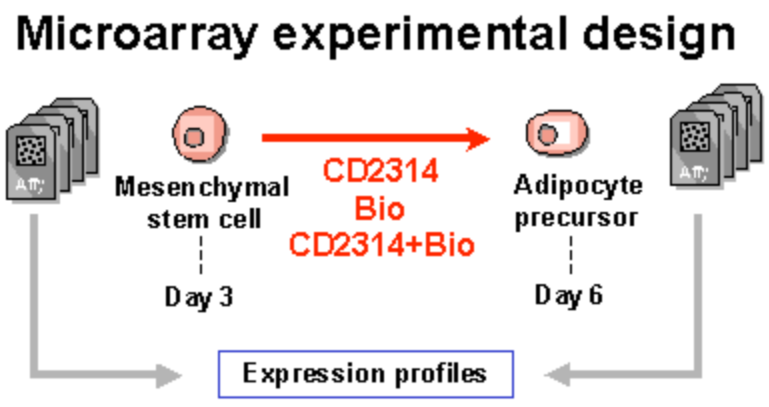

We have generated a high number of hanging drops, i.e., 62,100 drops, for each series of experiments and three independent series have been performed.

RNAs have been isolated and controlled for quality. Then, regulation of mesodermal differentiation for each condition and each series of experiments has been investigated by RT-PCR.

Abbreviation	Description	Arrays
day03	CGR8 cells at day 3; not stimulated	3
day06-0	CGR8 cells at day 6; not stimulated	3
day06-bio	CGR8 cells at day 6; stimulated with BIO	3
day06-cd	CGR8 cells at day 6; stimulated with CD2314	3
day06-cd-bio	CGR8 cells at day 6; stimulated with CD2314 and bio	3
day11-0	CGR8 cells at day 11; not stimulated	3
day11-bio	CGR8 cells at day 11; stimulated with BIO	3
day11-cd	CGR8 cells at day 11; stimulated with CD2314	3
day11-cd-bio	CGR8 cells at day 11; stimulated with CD2314and BIO	3

CNRS-UMR-6543 raw data (Affymetrix cel files of type Mouse 430_2) have been stored in the EMBL EBI ArrayExpress database:

http://www.ebi.ac.uk/arrayexpress/experiments/E-TABM-668

Accession: E-TABM-668

Project IPK-1: Differentiation of ESC to multilineage progenitors and further to committed pancreatic progenitors

Cell lines: R1; R1-PAX4

Alexandra Rolletschek and Anna Wobus

Contact: Anna Wobus

Leibniz Institute (IPK)

Correnstr.3

06466 Gatersleben, Germany

Tel: +49 394 825256

Email: wobusam@ipk-gatersleben.de

Summary of the project

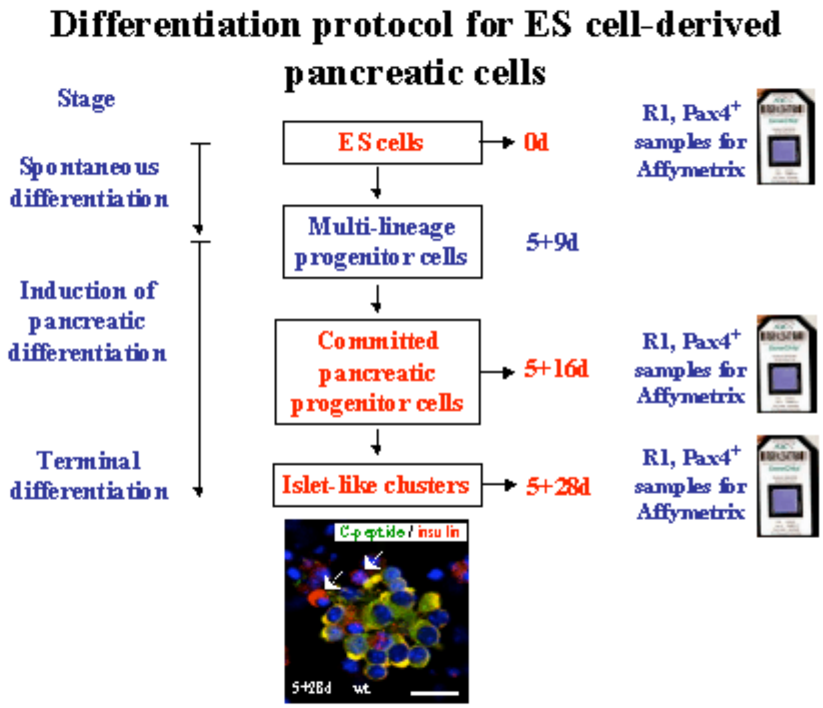

To characterize potential pancreatic progenitors during the process of induced pancreatic differentiation, wild-type R1 cells and R1 cells stably expressing the pancreatic control

gene Pax4, RNA samples of undifferentiated cells, pancreatic precursor and differentiated cells were prepared.

Abbreviation	Description	Arrays
R1-ESC-1	R1; ES cells (untreated)	5
R1-5-09d-2	R1; 5 + 9 days	5
R1-5-16d-3	R1; 5 + 16 days	5
R1-5-28d-4	R1; 5 + 28 days	5
Pax4-ESC-1	R1-PAX; ES cells (untreated)	5
Pax4-5-16d-3	R1-PAX; 5 + 16 days	5
Pax4-5-28d-4	R1-PAX; 5 + 28 days	5

IPK-1 raw data (Affymetrix cel files of type Mouse 430_2) have been stored in the EMBL EBI ArrayExpress database:

http://www.ebi.ac.uk/arrayexpress/experiments/E-TABM-493

Accession: E-TABM-493

Affymetrix GeneChip Analysis Central Facility

Herbert Schulz and Norbert Hübner

Contact: Norbert Hübner

Max-Delbrück-Center for Molecular Medicine (MDC) Berlin-Buch

Robert-Rössle-Str. 10

D-13092 Berlin, Germany

Tel: +49- 30 - 9406 2530

Email: nhuebner@mdc-berlin.de

Bioinformatics Team

Raivo Kolde, Priit Adler, Jüri Reimand and Jaak Vilo

Contact: Jaak Vilo

Institute of Computer Science, University of Tartu,
Liivi 2, 50409 Tartu, Estonia; and

Quretec, Ülikooli 6a, 51003 Tartu, Estonia

Tel: +372 50 49 365

Email: Jaak.Vilo@ut.ee

Additional FunGenES Principal Investigators

Michael Bader
Max-Delbrück-Center for Molecular Medicine (MDC)
Robert-Rösslestr. 10
D-13092 Berlin-Buch, Germany
Tel: +49-30-9406-2193 / 2119
Email: mbader@mdc-berlin.de

Frank Buchholz
Max-Planck-Institute of Molecular Cell Biology and Genetics
Pfotenhauer Str. 108
01307 Dresden, Germany
Tel: +49-351-2102888
Email: buchholz@mpi-cbg.de

Lesley Forrester
Queens Medical Research Institute, E2.47
University of Edinburgh
47 Little France Crescent
Edinburgh EH16 4TJ, UK
Tel: +44 131 242 9163
Email: L.Forrester@ed.ac.uk

Domingos Henrique
Instituto de Medicina Molecular
Faculdade de Medicina de Lisboa
Av. Prof. Egas Moniz
1649-028 Lisboa, Portugal
Tel: +351 21 7999516
Email: henrique@fm.ul.pt

Antonis K. Hatzopoulos

Department of Medicine and Department of Cell & Developmental Biology

Division of Cardiovascular Medicine

Vanderbilt University

2213 Garland Avenue - MRB IV P425C
Nashville, TN 37232-6300

Tel: +1-615-936 5529

Email: antonis.hatzopoulos@vanderbilt.edu

Heinz Himmelbauer

Max-Planck-Institute of Molecular Genetics

Ihnestr.73

D-14195 Berlin, Germany

Tel: +49-30-8413 1354

Email: himmelbauer@molgen.mpg.de
