# Supplementary figures and images for: The FunGenES Database: A Genomics Resource for Mouse Embryonic Stem Cell Differentiation
Source: PLoS One. 2009 Sep 3;4(9):e6804. doi: 10.1371/journal.pone.0006804 (PMC2731164; doi:10.1371/journal.pone.0006804)

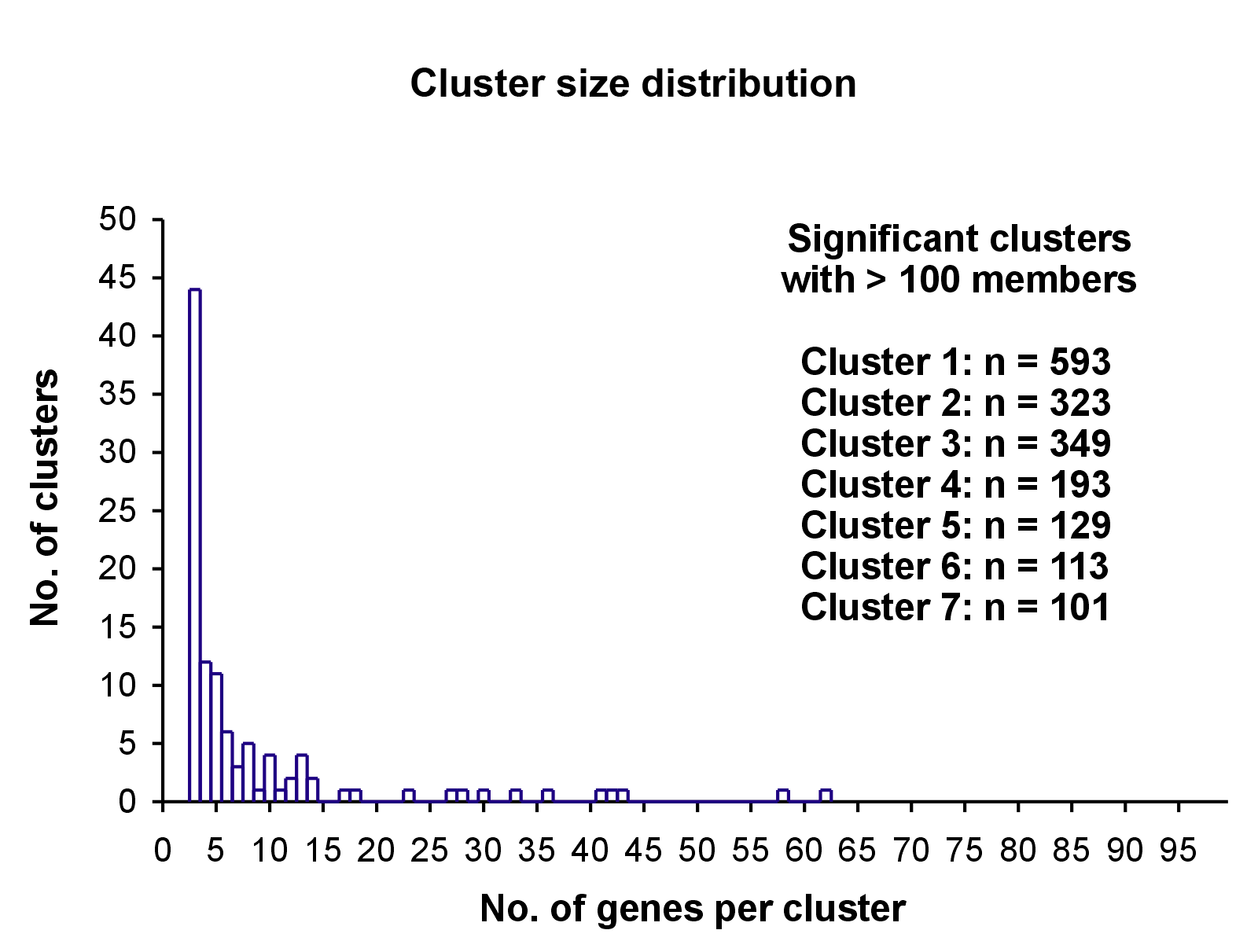

Supplement: Supplemental File S2 — Yield of the unsupervised hierarchical clustering. Histogram of the number of clusters (y-axis) for each cluster size (x-axis). Clusters with more than 100 genes are listed separately on the top right corner. (0.58 MB TIF) [file pone.0006804.s002.tif]

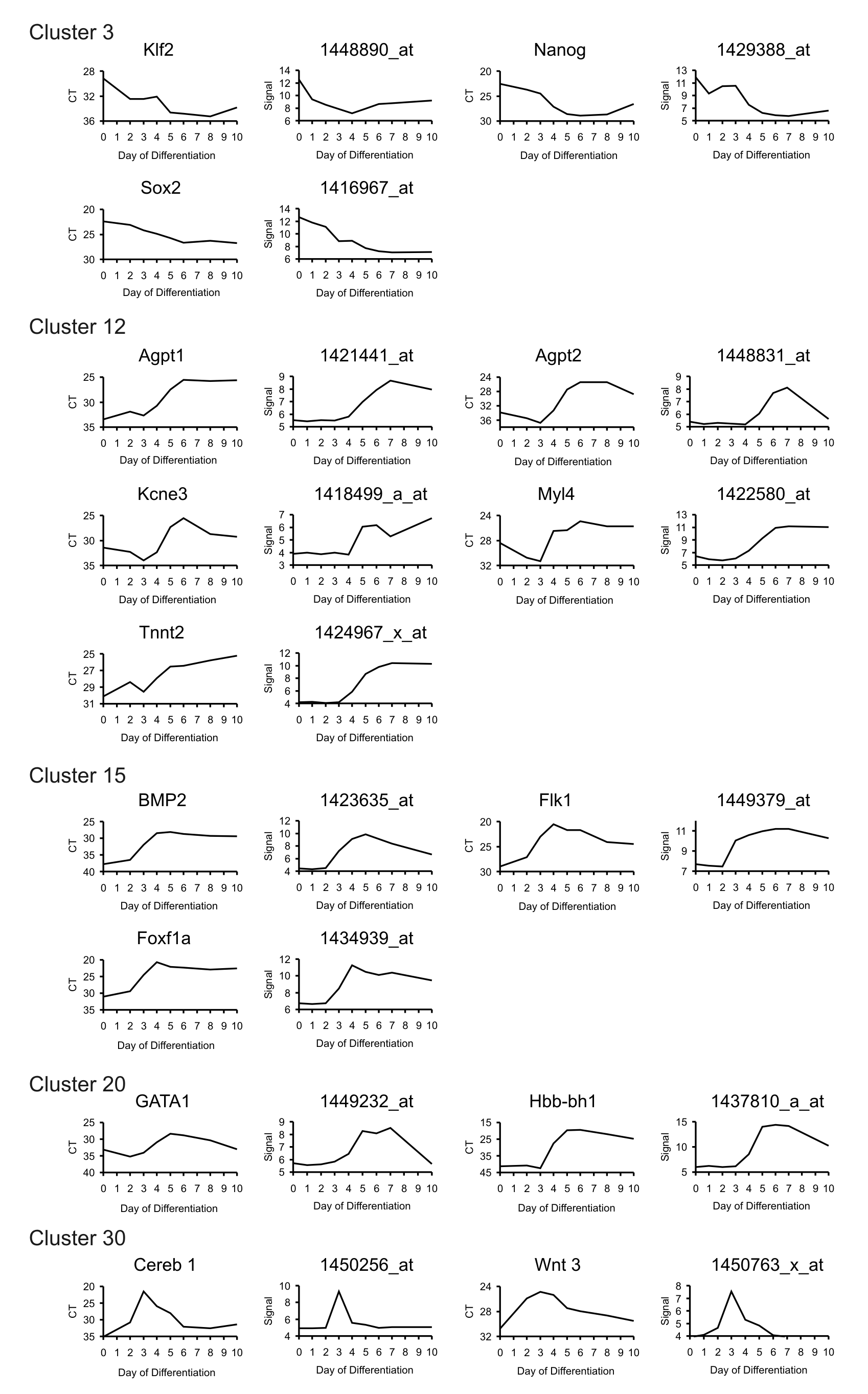

Supplement: Supplemental File S4 — Comparison of gene expression profiles obtained by Q-PCR (left panels) and microarray analysis (right panels). The gene name is depicted on top of the Q-PCR graph; the Affymetrix ID of the same gene marks the corresponding adjacent graph. CT: Cycle Threshold values of the Q-PCR analysis. Signal: normalized log2 signal values from the microarray analysis. Genes are organized according to the Global Cluster they belong as indicated. The results show comparable gene expression profiles between microarray and Q-PCR data. (0.97 MB TIF) [file pone.0006804.s004.tif]

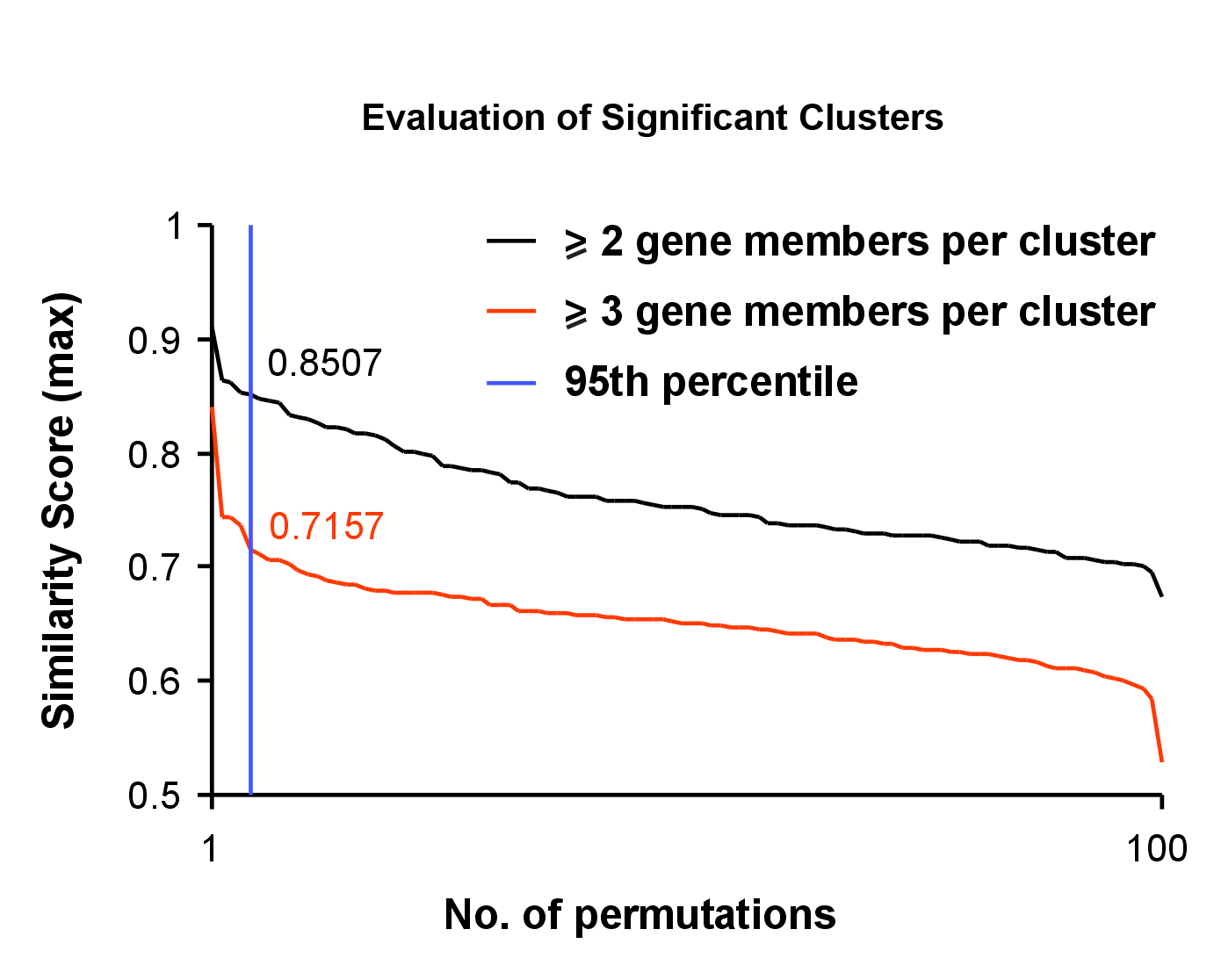

Supplement: Supplemental File S6 — Evaluation of significant correlations. Ranked plot of the best similarity scores (y-axis) of 100 permutated and clustered datasets (x-axis) and the evaluated 95th percentile of significant clusters (blue line). The results are given for cluster nodes with more than two (black line) or three (red line) cluster members. (0.55 MB TIF) [file pone.0006804.s006.tif]

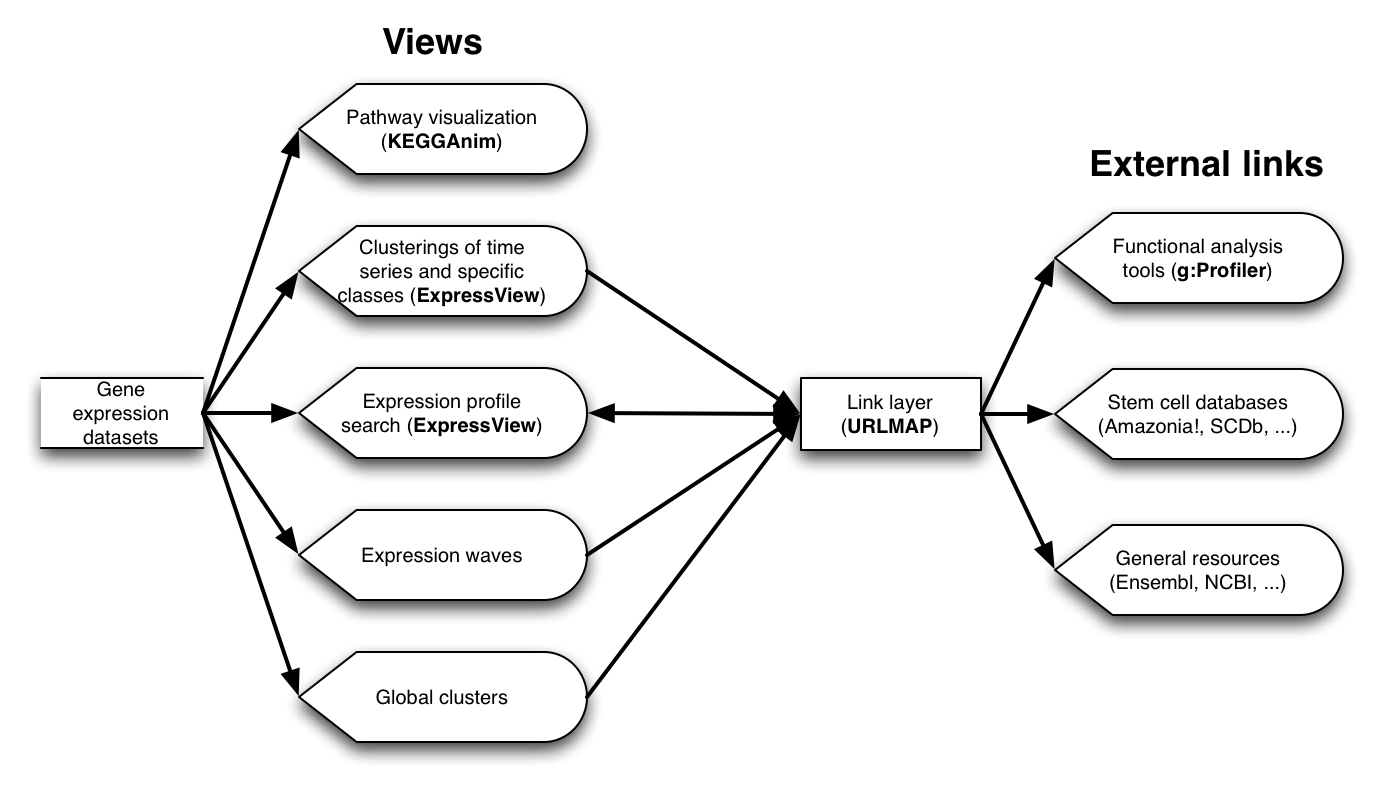

Supplement: Supplemental File S7 — Schematic representation of the FunGenES Database depicting tools to view expression data sets and links to external resources and databases. Tools in boldface have been developed specifically for the FunGenES Database. (0.79 MB TIF) [file pone.0006804.s007.tif]
