# Supplementary material for: The FunGenES Database: A Genomics Resource for Mouse Embryonic Stem Cell Differentiation
Source: PLoS One. 2009 Sep 3;4(9):e6804. doi: 10.1371/journal.pone.0006804 (PMC2731164; doi:10.1371/journal.pone.0006804)
Supplement: Supplemental File S5 — Experimental data sets used in Time Series (0.05 MB PDF) [file pone.0006804.s005.pdf]

Supplemental File S5

Experimental data sets used in Time Series

The samples from the 26 experimental conditions that represent untreated states without additional stimuli are listed below.

Experiment condition	Day of differentiation
CNRS-UMR-5164_h48liflif	0
AVEF-1_0es	0
IMBB-1_CGR8-LIF-TSA00	0
IPK-1_Pax4-ESC-1	0
IPK-1_R1-ESC-1	0
INS-1_Cre-ER-CTL	0
INS-1_STAT3-ER-CTL	0
INS-2_CGR8	0
INS-2_E14TG2a	0
INS-2_R1	0
UKOE-1_00d	0
UOB-1_DMSO	0
UOB-1_LY	0
CNRS-UMR-5164_h24---lif	1
UKOE-1_01d	1
UKOE-1_02d	2
CNRS-UMR-6543_day03	3
AVEF-1_eb3	3
UKOE-1_03d	3
AVEF-1_eb4	4
UKOE-1_04d	4
UKOE-1_05d	5
CNRS-UMR-6543_day06-0	6
AVEF-1_eb6ctl	6
UKOE-1_06d	6
UKOE-1_07d	7
